# Both Low and High PAPP-A Concentrations in the First Trimester of Pregnancy Are Associated with Increased Risk of Delivery before 32 Weeks in Twin Gestation

**DOI:** 10.3390/jcm9072099

**Published:** 2020-07-03

**Authors:** Aleksandra Saletra-Bielińska, Katarzyna Kosińska-Kaczyńska, Iwona Szymusik, Bartosz Kaczyński, Robert Brawura-Biskupski-Samaha, Szymon Kozłowski, Patrycja Jarmużek, Izabela Walasik, Mirosław Wielgoś

**Affiliations:** 1 Department of Obstetrics and Gynecology, Medical University of Warsaw, 02-091 Warsaw, Poland; asaletra@wum.edu.pl (A.S.-B.); iwona.szymusik@wum.edu.pl (I.S.); robertsamaha@gmail.com (R.B.-B.-S.); jarmuzek.patrycja@gmail.com (P.J.); miroslaw.wielgos@wum.edu.pl (M.W.); 2 Department of Obstetrics and Gynecology, Center of Postgraduate Medical Education, 01-809 Warsaw, Poland; 3 Department of Medical Informatics and Telemedicine, Medical University of Warsaw, 02-091 Warsaw, Poland; bartosz.kaczynski@wum.edu.pl; 4 University Center for Woman and Newborn Health of the Medical University of Warsaw, 02-015 Warsaw, Poland; Kozlowski1979@go2.pl; 5 Students Scientific Association at the 1st Department of Obstetrics and Gynecology, Medical University of Warsaw, 02-015 Warsaw, Poland; drzewicaiza@wp.pl

**Keywords:** preterm delivery, twin pregnancy, pregnancy associated plasma protein, perinatal outcome, intrauterine fetal demise

## Abstract

In twin gestation, the relationship between pregnancy associated plasma protein (PAPP-A) and perinatal outcome is unclear. The aim of the study was to determine if low and high concentrations of PAPP-A in the first trimester are related to perinatal outcome in twins. A retrospective study was conducted. Medical data of women in twin pregnancies who delivered between 2013 and 2018 were analyzed. PAPP-A concentrations were measured between 10 + 0 and 13 + 6 weeks. The associations between low (<10th percentile) and high (>90th percentile) values of PAPP-A and pregnancy complications were analyzed. A total of 304 patients were included. PAPP-A <10th percentile was associated with a high risk of preterm delivery (OR 6.14; 95% CI 2.1–18), delivery <34 weeks (OR 2.39; 95% CI 1.1–5.1) or <32 weeks (OR3.06; 95% CI 1.4–6.8). Significant relations between PAPP-A >90th percentile and delivery <34 weeks (OR4.09; 95% CI 1.8–9.1) or <32 weeks (OR 2.83; 95% CI 1.2–6.6) were found. PAPP-A >90th percentile was related to high risk of intrauterine fetal demise (OR 10; 95% CI 2.4–42.5). Both low and high PAPP-A concentrations seem to be related to pregnancy outcome. Further research is needed to investigate evaluation of risk of pregnancy complications according to PAPP-A concentrations as a continuous variable.

## 1. Introduction

Twins have a three to seven-fold higher risk of perinatal morbidity and mortality than singletons [1,2]. It is mainly due to preterm birth, which affects up to 66% of all twins [3]. Several factors have been found to be effective in predicting preterm delivery (PTD) in singletons, allowing to estimate the risk in the first, as well as in the second trimester of pregnancy. They include low concentration of pregnancy associated plasma protein (PAPP-A) in the first trimester, or sonographic measurement of cervical length in the second trimester of pregnancy [4]. PAPP-A is a glycoprotein produced by placental syncytiotrophoblast and decidua. It shows proteolytic activity for insulin-like binding proteins (IGFBP) 4 and 5, which play a role in the inhibition of insulin-like growth factors (IGF) 1 and 2. The IGF family has pleiotropic actions. It promotes cellular proliferation, differentiation and metabolism and therefore takes part in the control of placental and fetal growth [5]. PAPP-A increases the bioavailability of IGF 1 and 2, and is therefore involved in biological pathways promoting trophoblast invasion and vascularization of the placenta [4]. IGF 2 enables trophoblast invasion into the maternal decidua and glucose and amino acids transport into the villous cytotrophoblast [6].

In singletons, low concentration of PAPP-A in the first trimester is associated with an increased risk of small for gestational age (SGA), intrauterine demise (IUD), PTD or preeclampsia (PE) [4,7]. In twin gestations, the relationship between PAPP-A and perinatal outcome is not well established. The literature data are contradictory—there are both outcomes similar to singletons [8,9], as well as results showing no relationship between PAPP-A and perinatal outcome [10,11,12]. The aim of the study was to determine if low and high concentrations of PAPP-A in the first trimester of pregnancy are related to perinatal outcome in twins.

## 2. Materials and Methods

A retrospective study was conducted at the 1st Department of Obstetrics and Gynecology, Medical University of Warsaw, upon receiving the approval from the local ethic committee. Medical data of all women in twin pregnancies who received prenatal care at the outpatient clinic of the Department between 2013 and 2018 were analyzed. The inclusion criteria comprised of: diamniotic pregnancy, first trimester screening with PAPP-A level and nuchal translucency (NT) performed for all fetuses with a crown-rump length of 45 to 84 mm, and available information regarding the course of pregnancy, delivery and neonatal outcome. Both outpatient and hospital records were reviewed, in order to gain complete medical data. Monoamniotic pregnancies or those complicated by twin-to-twin transfusion syndrome, major anatomical anomalies of any of the fetuses, aneuploidy or lost to follow up gestations were excluded from the study.

Gestational age was calculated on the basis of the first day of the last menstrual period, or the transfer day in assisted reproduction techniques procedures and verified by the crown-rump length (CRL) measured in the first trimester (in case of CRL discordance, the measurement was taken from the larger twin). Women smoking cigarettes 5 years before the pregnancy and during pregnancy, regardless of the smoking cessation, were considered addicted to nicotine. PTD was defined as the delivery occurring before completed 37 weeks and very preterm delivery (VPTD) as one occurring before completed 32 weeks of gestation. Preterm premature rupture of membranes (PPROM) was defined as amniotic fluid leakage before 37 weeks of gestation, without spontaneous uterine contractions. Cervical insufficiency is defined as asymptomatic cervical shortening and dilatation with the absence of detectable uterine contractions. SGA newborn was a baby born with weight below the 10th percentile for gestational age, according to chorionicity [13]. Discordant twin growth was defined as twin birthweight difference exceeding 25% of the larger twin in each twin pair. Gestational hypertension (GH) and PE were diagnosed according to American College of Obstetricians and Gynecologists recommendations [14], whereas gestational diabetes mellitus (GDM), according to the Polish Society of Obstetricians and Gynecologists recommendations [15]. IUD was diagnosed by the death of a fetus after completed 22 weeks of gestation. The primary outcome of the study was VPTD, while secondary outcomes included the above-mentioned pregnancy complications (delivery below 37, 34 and 28 weeks GH, PE, GDM, IUD, SGA, discordant twin growth). 

All the patients were scheduled for sonographic examination at 11 + 0 to 13 + 6 weeks of gestation. PAPP-A concentrations were measured between 10 + 0 and 13 + 6 weeks. Kryptor (Brams AG) analyzer was used for biochemical measurements. Serum analytes were converted into multiples of median (MoM) and adjusted for gestational age, maternal weight and ethnicity. The associations between low and high biochemical values of PAPP-A MoMs (<10th percentile or >90th percentile) and study outcomes were analyzed.

Performed power analysis (Chi-squared test, proportions between two independent groups, Beta = 20%, alpha = 0.05) indicated that required sample size is 294. The expected VPTD in the group of 10–90th percentile PAPP-A was 11%, as previously published [3]. Variables were described as means with standard deviation or percentages. The Mann-Whitney test for continuous variables and Fisher’s exact test for qualitative data were used for statistical analysis. Odds ratios were estimated to study the association between biochemical values and study outcomes. The linearity of the associations was estimated using generalized additive models and visualized as spline functions. GAM R package (R 4.0.1 software) was used to create unrestricted spline functions. The best fitting of the smoothing spline was done using AIC criterion—knots position and numbers were automatically fitted to get best optimized curve (knots for presented spline functions are presented in Supplementary Table S1). General additive model using Cubic regression splines was used. *p* values <0.05 were considered significant. Data were analyzed using Statistica (version 13.3) and R software (R 3.5.2).

## 3. Results

A total of 304 patients met the inclusion criteria. The basic characteristics of the study population are shown in Table 1. A total of 46.7% of pregnancies were monochorionic. The mean gestational age at the time of the first trimester scan was 12 + 3 weeks. The percentages of abnormal analytes included 10.2% of cases with PAPP-A below the 10th percentile (0.49 MoM) and 9.2% with PAPP-A above the 90th percentile (1.84 MoM).

The relationships between PAPP-A concentrations and study outcomes are presented in Table 2. PAPP-A below the 10th percentile was associated with a significantly higher risk of PTD (RR 2.47; 95% CI 1.1–5.3), delivery <34 weeks (RR 2.25; 95% CI 1.1–4.6) and VPTD (RR 2.72; 95% CI 1.3–5.5). PAPP-A above the 90th percentile was also related to increased risk of delivery below 34 weeks (RR 3.46; 95% CI 1.6–7.5), 32 weeks (RR 2.48; 95% CI 1.1–5.3) and 28 weeks of gestation (RR 2.18; 95% CI 1.1–4.2). Both high and low PAPP-A increased the risk of VPTD over two-fold. No differences between the rates of cervical insufficiency or PPROM were noted between the groups. Spontaneous uterine contractions resulting in a delivery occurred significantly more often in the group of low PAPP-A (RR 2.05; 95% CI 1–4.2).

PAPP-A concentrations below the 10th percentile were associated with a more than three-fold increased risk of GDM (RR 3.5; 95% CI 1.7–6.9), while those above the 90th percentile were related to a significantly higher risk of IUD (RR 8.9; 95% CI 3.1–11.5). There were eight cases of IUD in the study group. Six of them occurred in monochorionic pregnancies, due to severe selective intrauterine growth restriction of one fetus, and two cases in dichorionic pregnancies: one due to intrauterine growth restriction and one of unknown etiology. No significant associations between PAPP-A concentrations and GH or PE, SGA or intertwin weight discordance were found.

Information on preterm delivery in monochorionic and dichorionic twins is presented in Table 3.

**Figure 1:**
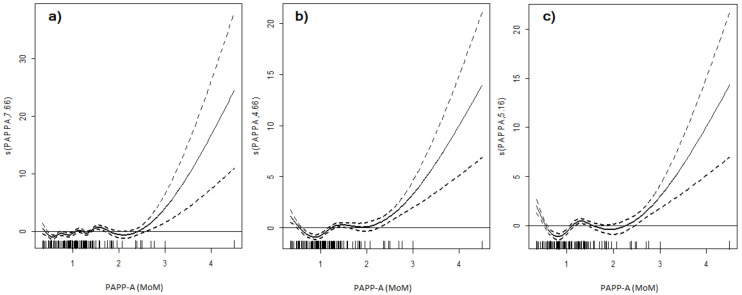
Generalized additive models of associations between PAPP-A concentration and preterm delivery (PTD) (**a**), delivery before 34 weeks (**b**) and delivery before 32 weeks of gestation (**c**) visualized as spline functions. (**a**): linear effect *p* < 0.001, non-linear effect *p* = 0.08; (**b**): linear effect *p* < 0.001, non-linear effect *p* < 0.001; (**c**): linear effect *p* < 0.001, non-linear effect *p* < 0.001. Vertical axis shows response values of logit (binomial) smooth spline function (“s” with name of the covariate, degrees of value). The bars in the bottoms of the figures present number of samples at each PAPP-A multiples of median (MoM) value.

Linear associations between study outcomes and PAPP-A concentrations were assessed using generalized additive models. A linear association between PTD and PAPP-A values was observed (linear effect *p* < 0.001; non-linear effect *p* = 0.08) and presented in Figure 1. 

Generally, the incidence of PTD increased in line with PAPP-A concentration. The spline function of the relation between PAPP-A MoMs and PTD was initially horizontal, and after a PAPP-A MoM value of about 2 was raised (knot 2.01). The general tendency was linear, and described by a significant linear effect of *p* < 0.001, and no non-linear effect was observed. Therefore, the spline function shows that generally there is a positive linear relation between PAPP-A MoMs and PTD, however, it is especially visible for values of PAPP-A MoM above 2. The spline function of the relation between PAPP-A MoMs and delivery before 34 weeks of gestation was linear as well (linear effect *p* < 0.001). However, another significant relation of a non-linear nature was also observed (non-linear affect *p* < 0.001). Therefore, the spline function of delivery before 34 weeks was U-shaped, meaning initially the risk of delivery below 34 weeks decreased with increasing values of PAPP-A (to PAPP-A 0.92 MoM value) and afterwards it increased with increasing values of PAPP-A (above PAPP-A value of 2.01). Both low and high values of PAPP-A MoMs were related to increased risk of delivery below 34 weeks of gestation. Analogous non-linear U-shaped spline function was observed for relation between PAPP-A values and VPTD (linear effect *p* < 0.001, non-linear effect *p* < 0.001; knots 0.92 and 2.07, respectively). The risk of delivery below 34 and 32 weeks decreased as the PAPP-A value increased (to 0.92 PAPP-A MoM), and afterwards increased again (above 2 PAPP-A MoM). Patients with PAPP-A around 0.92 MoM had the lowest risk of delivery before 34 (2/14—14.3%) and 32 weeks of gestation (1/14—7.1%).

## 4. Discussion

In our study, we found a significant relation between PAPP-A <10th percentile and a risk of preterm delivery (RR 2.47; 95% CI 1.1–5.3), delivery <34 weeks (RR 2.25; 95% CI 1.1–4.6) or <32 weeks (RR 2.72; 95% CI 1.3–5.5). On the other hand, another significant relation between PAPP-A >90th percentile and delivery <34 weeks (RR 3.46; 95% CI 1.6–7.5) or <32 weeks (RR 2.48; 95% CI 1.1–5.3) was observed. PAPP-A >90th percentile was also related to high risk of intrauterine fetal demise (RR 8.9; 95% CI 3.1–11.5). Both low and high PAPP-A concentrations were associated with a high risk of delivery before 34 and 32 weeks. 

The association between the first trimester aneuploidy biochemical markers and perinatal outcome in twins has been assessed by several authors and the reported results are confusing. We found a significant relation between PAPP-A concentrations and PTD, GDM and IUD risk. Iskender at al. analyzed perinatal outcome of 104 patients in twin gestation, and found no association between PAPP-A below the 10th percentile and SGA, PTD, GH or GDM, however, their study group was much smaller than ours [10]. A higher incidence of PTD in twin gestations with low PAPP-A (defined as below 0.42 MoM) was reported by Rosner et al. They analyzed the outcome of 340 patients, and found a significantly higher risk of PTD (RR 5.56; 95% CI 1.5–20.1) in women with low first trimester PAPP-A concentrations [8]. In another study by Laughon et al., the delivery prior to 32 weeks of gestation was almost three-fold more often in women with PAPP-A concentrations below the 25th percentile, though their results did not reach significance [9]. It is worth noticing that the study group in the research of Laughon et al. was also small (70 patients were included in the analysis). No association between low PAPP-A concentrations and PTD was reported by other authors either [11,12]. The performance of PAPP-A in prediction of PTD in twin gestation in several studies was assessed by Conde-Agudelo and Romero. Authors found the overall predictive ability of low serum levels of PAPP-A (defined as ≤25th percentile, <10th percentile, or <5th percentile) for preterm birth at <32, <34, <35, and <37 weeks of gestation to be minimal (sensitivities ranging from 5–56%, specificities from 78–95% and positive and negative likelihood ratios from 1.0–2.9 and 0.6–1.0, respectively) [16].

The above discrepancies may be related to the true complexity of relations between the risk of preterm delivery and PAPP-A concentrations. As the value of PAPP-A is a continuous variable, both linear and non-linear associations between perinatal outcomes and PAPP-A are possible. In the presented study, the associations between both low and high PAPP-A concentrations and delivery prior to 34 and 32 weeks of gestation were observed. A further analysis of relations between PAPP-A and preterm deliveries was conducted with the use of generalized additive models and both linear and non-linear relations were observed. A significant U-shaped association between PAPP-A concentration and the risk of delivery prior to 34 and 32 weeks was found. Therefore, researchers in future work should not focus on trying to identify single cut-off values of PAPP-A for determination of preterm delivery risk, as the relation between the two is both linear and non-linear in twin gestations.

Low concentrations of PAPP-A were found to be associated with abnormal placental function, SGA, IUD and PTD in singletons [4]. A correlation between PAPP-A concentration and trophoblast volume were observed [17]. As PAPP-A plays a significant role in regulation of IGF, and therefore takes part in the control of placental and fetal growth, and is related to placental function [5]. Observational studies suggested that the decrease in maternal serum PAPP-A concentrations in trisomic pregnancies is due to its posttranslational alteration. It may be related to impaired PAPP-A releasing mechanisms or reduced stability of the secreted protein [18]. Independently from the etiology of the decreased concentration, low PAPP-A causes downregulation of IGF 2 availability, which can lead to an impaired invasion of the trophoblast into maternal decidua, abnormal placentation in early pregnancy and impaired glucose and amino acids transport [19]. The above mechanisms may lead to spontaneous abortion or adverse perinatal outcome [6]. PAPP-A below the 5th percentile is related to increased risk of preterm delivery, fetal intrauterine growth restriction and preeclampsia [20]. According to Pelaez et al., placental lesions associated with placental malperfusion were seen more often in patients with twin gestation and low first trimester PAPP-A concentration [21]. The impairment of placentation and placental ischemia induced by the lower IGF bioavailability in early gestation may have an impact on the occurrence of PTD, although the true mechanism is not clear yet [5,19]. We found low PAPP-A concentration to be related to the risk of VPTD in twin gestation. On the other hand, we found high concentrations of PAPP-A to also be related to higher risk of delivery before 34 and 32 weeks. The mechanism of this relations is not known. As PAPP-A has a proteolytic activity on IGFBP, its elevated concentrations could lead to the decrease in IGFBP bioavailability. Wang et al. found significantly lower concentrations of maternal serum IGFBP 3 in women delivering singletons prior to 32 weeks of gestation, which may be due to high concentrations of PAPP-A [22]. It can be hypothesized that the elevated concentration of PAPP-A may have a similar effect on implantation and placentation as the decreased one. The desensitization of IGF receptors in the environment of excessive IGF release might be the possible mechanism; however, further studies are needed to confirm such theory. Another explanation may be that relatively bigger placental mass produce increased amount of PAPP-A. High PAPP-A may be related to fetal overgrowth, polyhydramnios and higher incidence of preterm delivery due to uterine overextension. In our study, no information on polyhydramnios or placental mass were available, while no differences between mean birthweight of the newborns or the occurrence of SGA between the groups of PAPP-A 10–90th percentile and >90th percentile were observed. Further studies evaluating the possible mechanisms of the relation between high PAPP-A and preterm delivery are needed.

We found significant association of elevated PAPP-A and the risk of IUD. No such relation had been described earlier in twins. Fathian et al. analyzed adverse outcomes in pregnancies with PAPP-A above 95th percentile and found no associations [11]. Our study is the first one reporting higher incidence of IUD in women with elevated first trimester PAPP-A. It can be assumed that the analogous mechanism as in preterm deliveries may be related to IUD in twin gestations with high PAPP-A concentrations.

The strength of the study is a numerous group of patients form a single clinic center. All patients were counselled according to the same local policy, and provided with the same medical standards. Clear definitions of prenatal complications were used for the whole group. A novel and unique analysis of the relationship between PAPP-A concentrations as a continuous variable and perinatal outcome was conducted. The presented study was the first one to estimate the association between the risk of IUD and PAPP-A concentration in twin gestation. The weakness of the study is its retrospective observational design. As both monochorionic and dichorionic pregnancies were included the study group may be inhomogeneous. It could bring additional information if a separate analysis could be made for iatrogenic and spontaneous PTD, however, this information was lacking. 

## 5. Conclusions

PAPP-A concentrations seem to be related with pregnancy outcome. Both low and high concentrations are associated with increased risk of delivery before 34 and 32 weeks of gestation. PAPP-A is routinely assessed in first trimester screening for aneuploidies and, therefore, it would be cost-effective as a prognostic toll in PTD and IUD risk assessment as well. Identifying women at a high risk of PTD or delivery before 32 weeks could help guide proper management and possible interventions. Further research is needed to investigate the exact mechanism of association between PAPP-A and PTD or IUD and novel evaluation of risk of pregnancy complications, according to PAPP-A concentrations as a continuous variable.

## Figures and Tables

**Table 1:** Basic characteristics of the study group.

	Study Group *n* = 304	PAPP-A <10th pc *n* = 31	PAPP-A 10–90th pc *n* = 245	*p*	PAPP-A >90th pc *n* = 28	*p*
	Means ± SD /*n* (%)	Means ± SD /*n* (%)	Means ± SD /*n* (%)		Means ± SD /*n* (%)	
age (years) *	34.02 ± 3.06	33.51 ± 4.1	34.12 ± 3.41	0.6	30.91 ± 3.89	0.08
Primiparity **	168 (55.3)	18 (58.1)	136 (55.51)	0.8	14 (50)	0.7
Monochorionicity **	142 (46.7)	16 (51.62)	108 (44.08)	0.4	18 (64.28)	0.047
BMI (kg/m^2^) *	22.94 ± 2.56	23.7 ± 1.62	22.97 ± 1.98	0.7	22.01 ± 2.25	0.8
Smoker **	17 (5.6)	3 (9.7)	14 (5.71)	0.4	0	0.3
ART **	50 (16.4)	2 (6.5)	46 (18.78)	0.1	2 (7.14)	0.2
gestational age at delivery (weeks) *	34.98 ± 3.08	32.65 ± 1.37	35.12 ± 3.23	0.03	32.82 ± 1.57	0.04
1st twin birtweight (g) *	2383 ± 582	2309 ± 378	2415 ± 498	0.1	2366 ± 404	0.2
2nd twin birtweight (g) *	2289 ± 538	2345 ± 214	2278 ± 642	0.3	2054 ± 225	0.08
1st twin SGA **	19 (6.3)	2 (6.5)	17 (6.94)	1	0	0.3
2nd twin SGA **	25 (8.2)	6 (19.35)	17 (6.94)	0.03	2 (7.14)	1

PTD—preterm delivery; VPTD—very preterm delivery; ART—assisted reproduction techniques; BMI—body mass index; SGA—small for gestational age newborn; *—Mann-Whitney test; **—Fisher’s exact test.

**Table 2:** Relationships between study outcomes and pregnancy associated plasma protein (PAPP-A) concentrations.

	PAPP-A <10th pc *n* (%)	PAPP-A 10–90th pc *n* (%)	*p*	OR (95% CI)	RR (95% CI)	PAPP-A >90th pc *n* (%)	*p*	OR (95% CI)	RR (95% CI)
Delivery < 37 weeks	28 (87.5)	130 (53.3)	<0.001	3.14 (2.1–18)	2.47 (1.1–5.3)	20 (71.4)	0.07	2.19 (0.9–5.2)	2.05 (0.9–4.9)
Delivery < 34 weeks	14 (43.8)	60 (24.6)	0.2	2.39 (1.1–5.1)	2.25 (1.1–4.6)	16 (57.1)	0.001	4.09 (1.8–9.1)	3.46 (1.6–7.5)
Delivery < 32 weeks	12 (37.5)	40 (16.4)	0.005	3.06 (1.4–6.8)	2.72 (1.3–5.5)	10 (35.7)	0.02	2.83 (1.2–6.6)	2.48 (1.1–5.3)
Delivery < 28 weeks	4 (12.5)	26 (10.7)	0.7	1.2 (0.4–3.7)	1.22 (0.4–3.3)	8 (28.6)	0.01	3.35 (1.3–8.4)	2.18 (1.1–4.2)
PPROM	5 (16.1)	26 (10.6)	0.4	1.6 (0.6–4.6)	1.52 (0.5–3.7)	4 (14.3)	0.7	0.6 (0.1–2.9)	1.35 (0.4–3.6)
Spontaneous uterine contractions resulting in delivery	10 (32.3)	42 (17.1)	0.049	2.3 (1–5.2)	2.05 (1–4.2)	7 (25%)	0.3	1.6 (0.6–4)	1.5 (0.6–3.5)
Cervix insufficiency	0 (0)	3 (1)	0.8	0.1 (0.1–4.2)	0.21 (0–6.4)	0 (0)	0.9	0.23 (0.1–3.1)	0.2 (0–3.8)
GDM	12 (37.5)	30 (12.3)	0.001	4.28 (1.9–9.6)	3.5 (1.7–6.9)	6 (21.4)	0.2	1.9 (0.7–5.2)	1.79 (0.6–4.2)
GH and PE	8 (25)	36 (14.7)	0.2	1.93 (0.8–4.6)	1.84 (0.6–3.9)	2 (7.1)	0.4	1.44 (0.1–1.9)	3.52 (1.7–6.9)
IUD	0	4 (1.6)	1	-	0 (0–8.6)	4 (14.3)	0.005	10 (2.4–42.5)	8.9 (3.1–11.5)
SGA	8 (25)	34 (13.9)	0.1	2.1 (0.9–4.9)	1.91 (0.8–4.1)	2 (7.1)	0.5	0.47 (0.1–2.1)	0.51 (0.1–2)
>25% BW	6 (18.8)	24 (9.8)	0.1	2.11 (0.8–5.7)	1.91 (0.7–4.4)	4 (14.3)	0.5	1.53 (0.5–4.8)	1.46 (0.4–3.9)

All Fisher’s exact test analyses; pc—percentile; PPROM—preterm premature rupture of membranes; GDM—gestational diabetes mellitus; GH—gestational hypertension; PE—preeclampsia; IUD—intrauterine fetal demise; SGA –small for gestational age newborn; >25% BW—intertwin birthweight discordance > 25%; OR—odds ratio; 95% CI—95% confidence interval.

**Table 3:** Relationships between preterm delivery and PAPP-A concentrations in monochorionic and dichorionic twins.

	Monochorionic *n* = 142			Dichorionic *n* = 162					
	PAPP-A <10th pc *n* = 15	PAPP-A 10–90th pc *n* = 114	PAPP-A >90th pc *n* = 13	PAPP-A <10th pc *n* = 16	*p* *	PAPP-A 10–90th pc *n* = 131	*p* **	PAPP-A >90th pc *n* = 15	*p* ***
delivery < 37 weeks	13 (86.67)	68 (59.65)	8 (61.54)	15 (93.75)	0.5	62 (47.33)	0.06	12 (80)	0.3
delivery < 34 weeks	7 (46.67)	31 (27.19)	7 (53.85)	7 (43.75)	0.8	29 (22.14)	0.4	9 (60)	1
delivery < 32 weeks	5 (33.33)	21 (18.42)	4 (30.8)	7 (43.75)	0.7	19 (14.5)	0.5	6 (40)	0.7
delivery < 28 weeks	2 (13.33)	12 (10.53)	4 (30.77)	2 (12.5)	0.8	14 (10.69)	0.8	4 (26.67)	0.6

*—PAPP-A <10th pc monochorionic vs. dichorionic; **—PAPP-A 10–90th pc monochorionic vs. dichorionic; ***—PAPP-A >90th pc monochorionic vs. dichorionic.

## Supplementary Materials

The following are available online at https://www.mdpi.com/2077-0383/9/7/2099/s1, Table S1: Knots for spline functions presented in the paper.

## References

[1] Lopriore E., Stroeken H., Sueters M., Meerman R.J., Walther F., Vandenbussche F. (2008). Term perinatal mortality and morbidity in monochorionic and dichorionic twin pregnancies: A retrospective study. Acta Obstet. Gynecol. Scand..

[2] Hack K.E., Derks J.B., Elias S.G., Franx A., Roos E.J., Voerman S.K., Bode C.L., Koopman-Esseboom C., Visser G.H. (2008). Increased perinatal mortality and morbidity in monochorionic versus dichorionic twin pregnancies: Clinical implications of a large Dutch cohort study. BJOG.

[3] Kosińska-Kaczyńska K., Szymusik I., Bomba-Opoń D., Olejek A., Sławska H., Zimmer M., Pomorski M., Bręborowicz G., Drews K., Seremak-Mrozikiewicz A. (2016). Perinatal outcome according to chorionicity in twins—A Polish multicenter study. Ginekol. Pol..

[4] Gagnon A., Wilson R.D. (2008). SOCIETY OF OBSTETRICIANS AND GYNAECOLOGISTS OF CANADA GENETICS COMMITTEE. Obstetrical complications associated with abnormal maternal serum markers analytes. J. Obstet. Gynaecol. Can..

[5] Shin J.E., Shin J.C., Kim S.J., Lee Y., Park I.Y., Lee S. (2016). Early midtrimester serum insulin-like factors and cervical length to predict preterm delivery. Taiwan J. Obstet. Gynecol..

[6] Santolaya-Forgas J., De Leon J.A., Cullen Hopkins R., Castracane V.D., Kauffman R.P., Sifuentes G.A. (2004). Low pregnancy-associated plasma protein-a at 10(+1) to 14(+6) weeks of gestation and a possible mechanism leading to miscarriage. Fetal Diagn. Ther..

[7] Turner J.M., Kumar S. (2020). Low First Trimester Pregnancy-Associated Plasma Protein-A Levels Are Not Associated with an Increased Risk of Intrapartum Fetal Compromise or Adverse Neonatal Outcomes: A Retrospective Cohort Study. J. Clin. Med..

[8] Rosner J.Y., Fox N.S., Saltzman D., Klauser C.K., Rebarber A., Gupta S. (2015). Abnormal Biochemical Analytes Used for Aneuploidy Screening and Adverse Pregnancy Outcomes in Twin Gestations. Am. J. Perinatol..

[9] Laughon S.K., Rebarber A., Rolnitzky L., Fink L., Saltzman D.H. (2009). Decreased first-trimester maternal serum free-beta subunit human chorionic gonadotropin and preterm birth in twin gestations. Am. J. Perinatol..

[10] Iskender C., Tarım E., Çok T., Yalcınkaya C., Kalaycı H., Yanık F.B. (2013). Obstetrical complications associated with first-trimester screening markers in twin pregnancies. J. Obstet. Gynaecol. Res..

[11] Fathian A., Miller R., Wolf E. (2014). Analysis of first trimester markers, PAPP-A and free-βhCG, and adverse outcomes in twin pregnancies. Am. J. Obstet. Gynecol..

[12] Chasen S.T., Martinucci S., Perni S.C., Kalish R.B. (2009). First-trimester biochemistry and outcomes in twin pregnancy. J. Reprod. Med..

[13] Ghi T., Prefumo F., Fichera A., Lanna M., Periti E., Persico N., Viora E., Rizzo G., Società Italiana di Ecografia Ostetrica e Ginecologica Working Group on Fetal Biometric Charts (2017). Development of customized fetal growth charts in twins. Am. J. Obstet. Gynecol..

[14] American College of Obstetricians and Gynecologists (2019). ACOG Practice Bulletin No. 202: Gestational Hypertension and Preeclampsia. Obstet. Gynecol..

[15] Wender-Ożegowska E., Bomba-Opoń D., Brązert J., Celewicz Z., Czajkowski K., Gutaj P., Malinowska-Polubiec A., Zawiejska A., Wielgoś M. (2018). Standards of Polish Society of Gynecologists and Obstetricians in management of women with diabetes. Ginekol. Pol..

[16] Conde-Agudelo A., Romero R. (2014). Prediction of preterm birth in twin gestations using biophysical and biochemical tests. Am. J. Obstet. Gynecol..

[17] Mesdaghi-Nia E., Behrashi M., Saeidi A., Abedzadeh Kalahroodi M., Sehat M. (2016). Association between PAPP-A and placental thickness. Int. J. Reprod. Biomed..

[18] Brizot M.L., Hyett J.A., Mckie A.T., Bersinger N.A., Farzaneh F., Nicolaides K.H. (1996). Gene expression of human pregnancy-associated plasma protein-A in placenta from trisomic pregnancies. Placenta.

[19] Pummara P., Tongsong T., Wanapirak C., Sirichotiyakul S., Luewan S. (2016). Association of first-trimester pregnancy-associated plasma protein A levels and idiopathic preterm delivery: A population-based screening study. Taiwan J. Obstet. Gynecol..

[20] Morris R.K., Bilagi A., Devani P., Kilby M.D. (2017). Association of Serum PAPP-A Levels in First Trimester with Small for Gestational Age and Adverse Pregnancy Outcomes: Systematic Review and Meta-Analysis. Prenat. Diagn..

[21] Paelez L., Chasen S., Baergen R. (2008). Relationship between first trimester maternal serum PAPP-A levels and placental lesions in twin gestations. Am. J. Obstet. Gynecol..

[22] Wang H.S., Perry L.A., Kanisius J., Iles R.K., Holly J.M., Chard T. (1991). Purification and assay of insulin-like growth factor-binding protein-1: Measurement of circulating levels throughout pregnancy. J. Endocrinol..

