# Supplementary material for: Both Low and High PAPP-A Concentrations in the First Trimester of Pregnancy Are Associated with Increased Risk of Delivery before 32 Weeks in Twin Gestation
Source: J Clin Med. 2020 Jul 3;9(7):2099. doi: 10.3390/jcm9072099 (PMC7408854; doi:10.3390/jcm9072099)
Supplement: Supplementary file 1 [file jcm-09-02099-s001.pdf]

Supplementary Table 1: Knots for spline functions presented in the paper**.**

Generalized additive models of associations between PAPP-A concentration and delivery <37 weeks	Knots: 0.31; 0.54; 0.77; 0.98; 1.23; 1.47; 1.93; 2.01; 2.18; 2.45 PAPP-A MoM
Generalized additive models of associations between PAPP-A concentration and delivery <34 weeks	Knots: 0.51; 0.75; 0.92; 1.37; 1.49; 1.89; 2.01; 2.33; 3.23 PAPP-A MoM
Generalized additive models of associations between PAPP-A concentration and delivery <32 weeks	Knots: 0.45; 0.78; 0.92; 1.24; 1.33; 1.74; 2.07; 2.37; 3.02; 3.42 PAPP-A MoM
